# Environmental Enrichment Partially Repairs Subcortical Mapping Errors in Ten-m3 Knock-Out Mice during an Early Critical Period

**DOI:** 10.1523/ENEURO.0478-18.2019

**Published:** 2019-12-03

**Authors:** Peta Eggins, James Blok, Justin Petersen, Larissa Savvas, Lara Rogerson-Wood, Hannan Mansuri, Atomu Sawatari, Catherine A. Leamey

**Affiliations:** Discipline of Physiology, School of Medical Sciences and Bosch Institute, Faculty of Medicine and Health, University of Sydney, New South Wales 2006, Australia

**Keywords:** axon guidance, binocular, dLGN, ipsilateral, plasticity, teneurin/Ten-m/Odz

## Abstract

Environmental enrichment (EE) has been shown to improve neural function via the regulation of cortical plasticity. Its capacity to induce functional and/or anatomical repair of miswired circuits is unknown. Ten-m3 knock-out (KO) mice exhibit a highly stereotyped and profound miswiring of ipsilateral retinogeniculate axons and associated deficits in binocularly-mediated visual behavior. We determined whether, and when, EE can drive the repair of subcortical wiring deficits by analyzing Ten-m3 KO and wild-type (WT) mice that were enriched for six weeks from adulthood, weaning or birth in comparison to standard-housed (SE) controls. Six weeks of EE initiated from birth, but not later, induced a significant reduction in the area occupied by ipsilateral retinogeniculate terminals in KOs. No EE-induced correction of mistargeted axons was observed at postnatal day (P)7, indicating that this intervention impacts pruning rather than initial targeting of axons. This reduction was most prominent in the ventrolateral region of the dorsal lateral geniculate nucleus (dLGN), suggesting a preferential pruning of the most profoundly mistargeted axons. EE can thus partially repair a specific, subcortical axonal wiring deficit, but only during an early, developmentally-restricted time window.

## Significance Statement

The incorrect wiring of neural circuits can lead to profound disability. Using a mouse model which exhibits a marked miswiring of neural projections to subcortical relay centers of the brain, we show that positive modulation (enrichment) of the environment during the first few postnatal weeks can induce the pruning of aberrant neural projections. The capacity for environmental enrichment (EE) to correct miswired projections diminished before adolescence, suggesting that the enrichment can only drive these changes during an early critical period. This expands our knowledge of the capacity for enrichment to induce repair of neural circuits. The demonstration of dramatic effects on targeting of neural projections over a restricted period may have implications for the development of therapies for neurodevelopmental disorders.

## Introduction

There is compelling evidence that the mature patterns of neural circuitry depend on interactions between an intrinsic genetic scaffold and patterns of experience-dependent activity which sculpt these connections (Sur and Leamey, 2001). An ongoing issue is to determine how the interactions between these factors may be regulated in a manner which maximizes functional ability. This holds enormous promise for the development of non-invasive therapies for a large range of neural disorders (Sale et al., 2014; Rogers et al., 2019).

Classic studies performed using the model of the mammalian visual cortex in the mid-20th century illustrated the importance of activity in sculpting neural connectivity (Wiesel and Hubel, 1963a,b; Hubel et al., 1977). These, and other works (for review, see Hensch and Quinlan, 2018), established the notion that the balance of activity patterns arising from the eyes is crucial during an early postnatal critical period when the cortex is highly plastic, and that cortical plasticity subsequently declines. More recent studies have shown, however, that positive modulation of the environment can render a powerful influence on cortical plasticity outside of this early window. Notably, exposure of laboratory animals to more complex social and physical environments [termed environmental enrichment (EE)] can induce epigenetic mechanisms which are thought to extend the capacity for high levels of cortical plasticity typical of juveniles well into adulthood (Sale et al., 2007; Baroncelli et al., 2010, 2016; Scali et al., 2012; Greifzu et al., 2016).

The discovery that experience can induce changes which modulate the capacity of the cortex to adapt to the environment is exciting, but raises other questions, including how generalizable these principles are: is EE able to induce plasticity sufficient to promote the recovery of circuits that are dysfunctional, not just due to the balance of excitatory and inhibitory weights of synaptic inputs, but via more dramatic changes in connectivity that result from impaired axonal guidance? Is the primary locus of repair limited to the cortex, or can EE induce the correction of miswired subcortical circuits? If so, is this capacity maintained across the life span of the animal, or is there a “critical period” for its influence?

The visual pathway of Ten-m3 knock-out (KO) mice presents an ideal model to begin addressing these questions. In wild-type (WT) mice, this pathway is highly stereotyped, and accessible to detailed anatomical and functional assessment. The Ten-m (teneurin/Odz) family of highly-conserved Type II transmembrane proteins can regulate axonal guidance, dendritic morphology, as well as synaptic structure and function (Leamey et al., 2007; Young and Leamey, 2009; Dharmaratne et al., 2012; Hong et al., 2012; Mosca et al., 2012; Antinucci et al., 2013, 2016; Young et al., 2013; Glendining et al., 2017; Berns et al., 2018). Crucially here, Ten-m3 KO mice display a dramatic and consistent miswiring of ipsilateral retinal projections within the dorsal lateral geniculate nucleus (dLGN; Leamey et al., 2007). Unlike WT mice, whose ipsilateral projections are confined to dorsomedial dLGN, in Ten-m3 KOs ipsilateral axons terminate in an elongated strip that extends far into ventrolateral dLGN (Leamey et al., 2007). The mapping deficits are transferred to the primary visual cortex (V1; Merlin et al., 2013). The resulting misaligned connectivity of ocular inputs leads to profound functional deficits (Leamey et al., 2007; Merlin et al., 2013).

We sought to determine whether EE was able to induce changes that would enable the correction of profoundly miswired axonal projections. Further, we wished to establish whether the sensitivity to EE in this circuit was maintained across the life span or was age dependent. The termination patterning of retinogeniculate axons of mice that had experienced six weeks of EE from birth (EE-B), weaning or adulthood were compared to age-matched standard-housed (SE) controls. Anterograde tracing revealed that six weeks of EE initiated from birth, but not later, induced a significant reduction of ipsilateral retinogeniculate terminals in Ten-m3 KOs; the most aberrant projections showed the greatest retraction. No evidence of EE-induced changes in the targeting of ipsilateral retinal axons could be seen in KO mice analyzed at one week of age. Together, these results indicate that the correction is due to enhanced pruning rather than improved axonal targeting during ingrowth. We conclude that EE is able to induce a substantial, if partial, correction of miswired retinal outputs to subcortical targets. In contrast to what has been shown for the visual cortex, however, EE can only induce changes in this aberrant subcortical wiring during an early developmental time window.

## Materials and Methods

The protocol was approved by Animal Ethics Committee of the University of Sydney and conformed to guidelines of the Society for Neuroscience’s policy on animal use as well as those of the National Health and Medical Research Council of Australia. All animals were housed in climate-controlled rooms (∼23.5°C, 40–70% humidity) at the Institutional Animal Housing Facility on a fixed 12/12 h light/dark cycle. Standard mouse chow and water were provided *ad libitum*.

### Animals

Ten-m3 KO and WT mice were obtained by breeding female heterozygotes with male heterozygotes in standard cages (see Standard and enriched housing). Mice were genotyped using tissue biopsy followed by polymerase chain reaction (Leamey et al., 2007). Mice of both genders were included in the study in approximately equal numbers.

### Standard and enriched housing

Animals raised in standard conditions were housed in individually ventilated plastic cages (32.5 × 15 × 16.5 cm). Each cage housed two to five mice and contained shredded paper for nesting, an igloo, food hopper, and water bottle.

Animals exposed to in EE were housed in large, two-story cages (45 × 37.5 × 39 cm). Each cage housed at least 3–10 mice and contained a mouse igloo with running wheel, one long and one short toilet paper roll, half a tissue box, three to five marbles, one to two ping pong balls, multicolored paddle pop sticks tied together with multicolored pipe cleaners, two high contrast visual stimuli (a checkerboard and a diagonal grating), and three scented plush ball toys. These objects were chosen to stimulate as many senses as possible; the running wheel provided access to voluntary exercise. The positions of enrichment objects in the EE cages were changed three times a week for added stimulation and replaced/re-scented as required.

Dams were either transferred to individual standard housing cages (one dam per cage), or in the case of EE-B in pairs into enrichment cages (two dams per cage), in the last 2–3 d of pregnancy. Pups were weaned into sex-specific cages at three weeks of age [postnatal day (P)21]. Pups allocated to commence EE from weaning (EE-W) were weaned from dams housed in standard conditions into EE cages. Mice allocated to commence EE in adulthood (EE-A) were transferred into sex-specific enrichment cages at three to six months of age. Mice from all three enrichment groups experienced EE conditions for six weeks (i.e., until they reached six weeks for EE-B, nine weeks for EE-W, and five to eight months for the EE-A groups).

For SE age-matched control animals, mice were bred in conventional cages. They were weaned at three weeks into sex-specific standard cages and raised until they reached the appropriate age for comparison with EE groups [six weeks for standard birth (SE-B), nine weeks for standard weaning (SE-W), and five to eight months for standard adult (SE-A) groups].

### Anatomical tracing

For anterograde tracing from the retina of both WT and KO mice, intraocular injections of 0.5–1 μl of 1% cholera-toxin subunit B (CTB) conjugated to either Alexa Fluor 488 or Alexa Fluor 594 were made into the vitreous chamber of each eye under isofluorane anesthesia at the appropriate age. After a 24- to 72-h transport period, mice were anesthetized with 2–4% isofluorane-oxygen mixture, euthanized (>100 mg/kg of sodium pentobarbitone), and perfused with 0.9% saline followed by 4% paraformaldehyde in 0.1 M phosphate buffer. Brains were postfixed at 4°C for at least 24 h and cryoprotected in 30% sucrose in 0.1 M phosphate buffer (pH 7.4). Coronal sections, 60 μm thick, were prepared using a freezing microtome, mounted on microscope slides, coverslipped, and examined.

### Microscopy and analysis

Sections were viewed and imaged using a wide-field fluorescence Zeiss Apotome 2 deconvolution microscope with Texas Red (excitation wavelength 596 nm; emission wavelength 613 nm) and Alexa Fluor 488 (excitation wavelength 490 nm; emission wavelength 525 nm) filters to visualize each tracer.

Four sections from the rostral third of the dLGN were selected from each animal (*n* = 5–8 animals per group), as it is in this region that the ventrolateral expansion of the ipsilateral terminals is reported to be most apparent (Leamey et al., 2007). The efficacy of the ipsilateral tracer injection was checked by examining the labeling distribution in the corresponding contralateral dLGN. Poorly labeled cases were excluded from the analysis. Contrast and brightness were adjusted uniformly to optimize images and analyzed using ImageJ (NIH). For this, background was subtracted using the rolling ball radius function and images were thresholded. The area occupied by thresholded ipsilateral label relative to the total area of the dLGN was measured for each section [mean and standard errors of the mean (s.e.m.) were calculated for all sections per housing (SE/EE) – age (birth, weaning, and adulthood) – genotype (WT/KO) group; e.g., SE-B WT; see text]. To determine any region-specific changes in ipsilateral retinogeniculate terminal labeling, the “long axis” of the dLGN (defined as the maximal length along the dorsomedial to ventrolateral (DM-VL) extent of the nucleus within the coronal section) was measured, and divided into three equidistant dorsal, middle, and ventral regions. The proportion of total ipsilateral label within each of these regions was then calculated (percentage of ipsilateral distribution).

The percentage area of the dLGN occupied by ipsilateral terminals was compared across groups using a univariate ANOVA with genotype, age, and housing condition as fixed factors, and sections included as a random factor. Differences between groups of interest were determined via pairwise testing, corrected for multiple comparisons (Bonferroni). Percentage of ipsilateral distribution by region values were analyzed using a univariate ANOVA with genotype, age, housing condition as fixed factors, and both section and region as random factors. Bonferroni corrected pairwise comparisons were used to reveal specific differences. For P7 measurements, percentage area of dLGN occupied by ipsilateral terminals was compared using a univariate ANOVA with housing as fixed, and sections as random factors, as above. For percentage of ipsilateral distribution by region values, differences were assessed using a univariate ANOVA with housing as fixed, and region as random factors. (Sections were not able to be included as a random factor in this analysis as the small error prevented the error degrees of freedom for housing from being calculated accurately. Housing effects were assessed either by considering housing and section as fixed and region as random factors, or by setting housing as fixed and region as random factors. The outcomes of these different approaches were identical and so only the latter is reported.) A significance value of α = 0.05 was assumed for all statistical tests.

## Results

### Enrichment from birth, but not from weaning or adulthood, drives a partial retraction of miswired ipsilateral retinogeniculate projections

**Figure 1.**
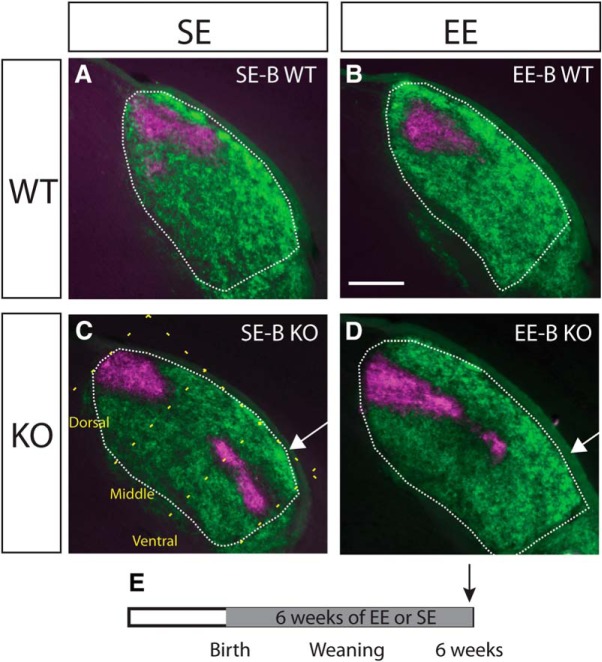
Mapping of ipsilateral retinogeniculate projections is partially corrected in Ten-m3 KOs enriched for six weeks from birth. Coronal sections through the rostral half of the dLGN (outlined) of mice following injections of anterograde fluorescent tracers into both eyes (dorsal is to the top and lateral to the right in all sections). Top row (***A***, ***B***) shows examples from standard-housed and enriched WTs from the birth group (SE-B WT and EE-B WT, respectively). Ipsilateral retinal projections are confined to a patch in the dorsomedial dLGN (magenta), while the contralateral projections (green) fill the rest of the nucleus. There are no obvious housing-related changes in WTs. An example from an SE-B KO is shown in ***C***. Ipsilateral projections are elongated along the dorsomedial-ventral axis of the dLGN. An arrow highlights the ipsilateral terminal expansion into the visuotopically inappropriate far ventrolateral region of the dLGN. The dotted yellow line in ***C*** illustrates the dorsomedial-ventrolateral axis of the dLGN, as well as the orthogonal divisions that divided the dLGN into thirds along this axis. These divisions were used in the quantitative analysis shown in Figure 4. Panel ***D*** provides an example from an EE-B KO. Ipsilateral terminals are markedly less elongated along the DM-VL axis of the dLGN compared to SE-B KOs. Notably, there is very little label in the ventral region of the dLGN (***D***; arrow). ***E***, Schematic diagram indicating the time course of the experimental paradigm. The gray region indicates the period of exposure to EE versus SE. The arrow indicates the point of analysis. Scale bar in ***B*** = 200 μm, applies to all images.

We first investigated the impact of exposure to six weeks of EE-B on the distribution of ipsilateral terminals using anterograde tracing of retinogeniculate projections with fluorescent CTB. The expected distribution of label in all standard-housed birth group (SE-B) WTs was observed, with ipsilateral projections confined to the dorsomedial region of the dLGN (Fig. 1*A*). There was no obvious impact of EE-B on the patterning of ipsilateral terminals in WTs (Fig. 1*B*). All SE-B KOs exhibited a narrowed and elongated distribution of the ipsilateral label which expanded into the ventrolateral region of nucleus (Fig. 1*C*). In Ten-m3 KOs enriched from birth, however, ipsilateral projections were noticeably less elongated along the dorsomedial-ventrolateral axis of the dLGN (Fig. 1*D*) compared to SE-B KOs suggesting an effect of EE, although the patterning still differed qualitatively from terminal distributions observed in WTs.

**Figure 2.**
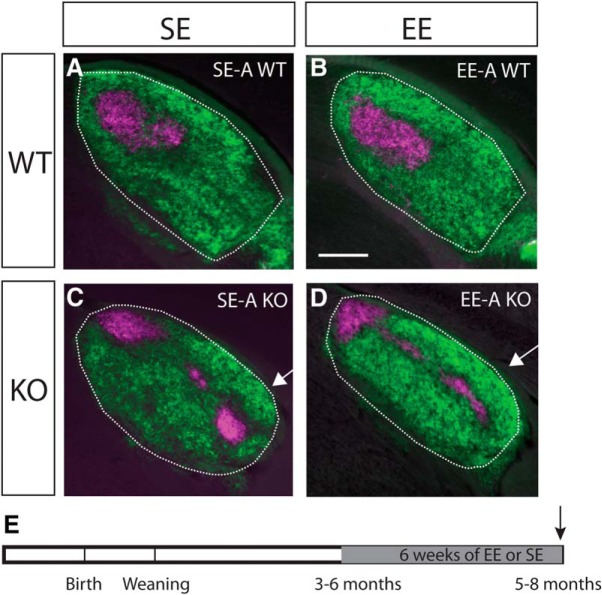
EE during adulthood does not impact targeting of ipsilateral retinogeniculate projections. Conventions as for Figure 1, showing examples from mice enriched during adulthood (EE-A) compared to standard-housed controls (SE-A). Coronal sections through the rostral half of the dLGN (outlined) of mice following injections of anterograde fluorescent tracers into both eyes (dorsal is to the top and lateral to the right in all sections). No effect of EE on the targeting of ipsilateral retinal projections was apparent in WTs (***A***, ***B***) or Ten-m3 KOs (***C***, ***D***). Note ipsilateral label extends into far ventrolateral dLGN in both EE-A-KOs and SE-A KOs (arrows in ***C***, ***D***). ***E***, Schematic diagram indicating the time course of the experimental paradigm. The gray region indicates the period of exposure to EE versus SE. The arrow indicates the point of analysis. Scale bar in ***B*** = 200 μm, applies to all images.

We then asked whether the impact of EE retinogeniculate projections is maintained into adulthood. In Ten-m3 KOs enriched during adulthood (EE-A; Fig. 2*D*), no change in the distribution of ipsilateral retinal terminals was observed compared to standard-housed adult controls (SE-A; Fig. 2*C*). WTs also showed no changes with EE during adulthood (Fig. 2*A*,*B*). This suggests that there may be a critical period for the impact of EE on miswired axonal projections.

**Figure 3.**
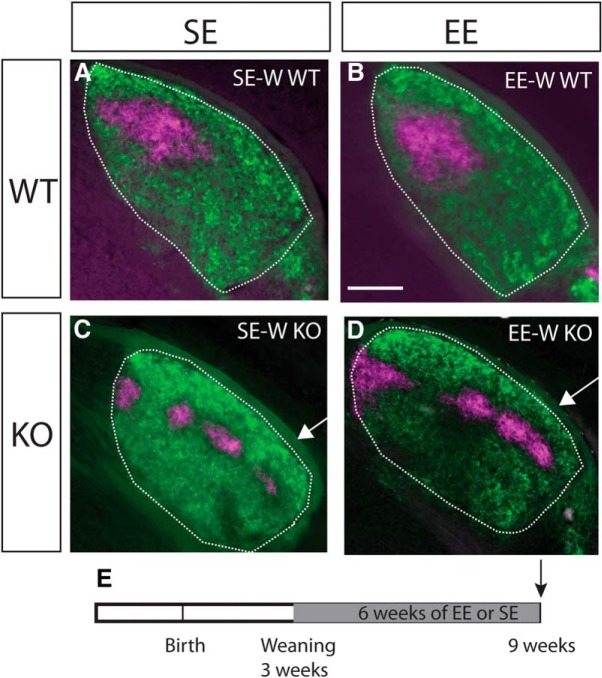
EE-W does not impact targeting of ipsilateral retinogeniculate projections. Conventions are as for Figure 1, but showing examples from mice enriched from weaning (EE-W) compared to standard-housed controls (SE-W). Coronal sections through the rostral half of the dLGN (outlined) of mice following injections of anterograde fluorescent tracers into both eyes (dorsal is to the top and lateral to the right in all sections). No effect of EE on the targeting of ipsilateral retinal projections was apparent in WTs (***A***, ***B***) or Ten-m3 KOs (***C***, ***D***). Note ipsilateral label extends into far ventrolateral dLGN in EE-W KOs and SE-W KOs (arrows in ***C***, ***D***), similar to what was observed in adult enriched samples. ***E***, Schematic diagram indicating the time course of the experimental paradigm. The gray region indicates the period of exposure to EE versus SE. The arrow indicates the point of analysis. Scale bar in ***B*** = 200 μm, applies to all images.

To narrow down the timing of sensitivity to EE, we exposed mice to six weeks of EE from weaning (P21). This did not result in any obvious changes compared to standard-housed age-matched controls (SE-W) in either WTs (Fig. 3*A*,*C*) or KOs (Fig. 3*B*,*D*).

**Figure 4.**
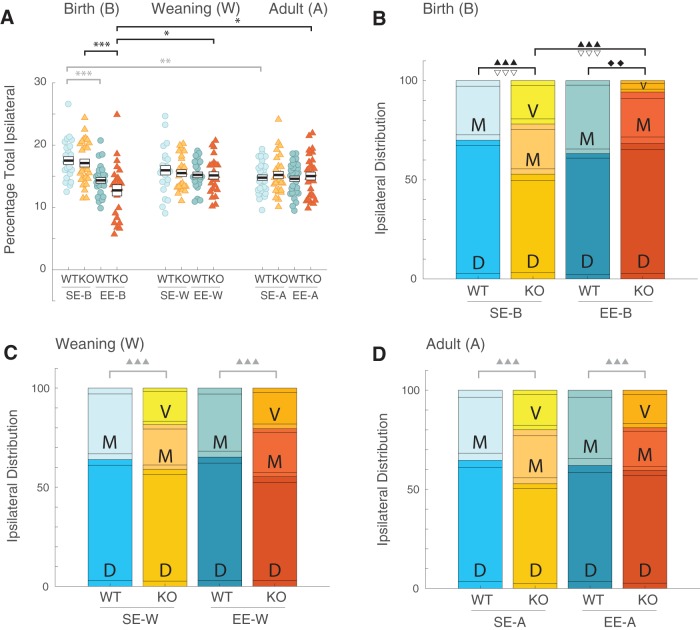
Enrichment from birth leads to a loss of ipsilateral retinal inputs to ventral dLGN in Ten-m3 KOs. Graphs show quantification of total ipsilateral inputs and their relative distribution in the dLGN. Data derived from individual animals in each group is shown. Thick black lines show group means, boxes indicate s.e.m.. Housing groups are separated by age with the birth cohort shown on the left, weaning groups in the middle, and adults on the right. The same shading and color coding for housing; lighter shade for standard-housing groups (SE), darker for all EE groups, and genotype (blue for WT; red for KO) is used across age groups for ease of comparison. All SE WTs are shown in light blue and all EE WTs are shown in darker blue. All SE KOs are shown in orange, and all EE KOs are shown in red. ***A***, Percentage of total dLGN occupied by ipsilateral terminals. A slight but significant decrease was seen between the SE-B WTs versus the EE-B WTs (gray ***p < 0.001). A decrease of greater magnitude was detected between SE-B KOs and EE-B KOs (black ***p < 0.001), consistent with the decreased expanse of ipsilateral terminals observed qualitatively in the enriched from birth cohort. A small but significant decrease was also observed between the SE-B WTs and SE-A WTs (gray **p = 0.003), suggesting that pruning of retinogeniculate terminals continues well after adolescence in standard-housed WTs. EE-B KO values were significantly smaller than EE-W KOs (*p = 0.021) and EE-A KOs (*p = 0.023), indicating that the timing of enrichment had a major impact on pruning of ipsilateral projections. No effects of housing or genotype on total ipsilateral area were seen within the weaning or adult groups. ***B–D***, Relative distributions of ipsilateral terminals across the dorsal (D), middle (M), and ventral (V) thirds of the dLGN for birth (***B***), weaning (***C***), and adult (***D***) SE and EE cohorts (see Materials and Methods). Stacked bars show mean values (large single-color boxes) plus s.e.m. (smaller flanking boxes of the same color, present on both top and bottom of each mean box) for proportion of ipsilateral terminals present in D, M, and V regions. Within WTs, there was no detectable change in distribution across regions of ipsilateral terminals between ages or housing conditions. No terminals were found in the ventral third of the dLGN in WTs. SE-B KOs had significantly less label in dorsal dLGN (▿▿▿*p* < 0.001) and significantly more label in ventral dLGN (▴▴▴*p* < 0.001) than SE-B WTs. In EE-B KOs, there was a significant increase in the percentage of terminals in the dorsal region (▿▿▿*p* < 0.001) along with a significant decrease in the ventral region (▴▴▴*p* < 0.001), compared to SE-B KOs. No difference in the proportion of ipsilateral terminals in dorsal (*p* = 0.158) as well as ventral (*p* = 0.112) dLGN was observed between EE-B KOs and EE-B WTs. A detectable decrease in EE-B KOs relative to EE-B WTs was also present for the middle region (⧫⧫*p* = 0.003). Weaning and adult groups showed differences in the ventral region with respect to genotype but not housing (KOs > WTs: 
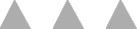
*p* < 0.001 in ***C***, ***D***).

To quantitatively assess these effects, the total area of ipsilateral terminal labeling was measured and expressed as a percentage of the total cross-sectional area of the dLGN (see Materials and Methods; Fig. 4*A*). Four sections from six to eight animals per group were quantified. These values were similar in SE-B WTs (mean ± s.e.m.: 17.52 ± 0.63%) and SE-B KOs (17.12 ± 0.58%). Interestingly, EE-B appeared to decrease the proportion of the dLGN occupied by the ipsilateral terminals in both genotypes, with a change of larger magnitude observed in KOs (EE-B WTs: 14.35 ± 0.58%; EE-B KOs: 12.74 ± 0.63; Fig. 4*A*, left). Neither genotype or EE impacted the percentage of the dLGN occupied by ipsilateral terminals in the weaning (SE-W WT: 15.96 ± 0.63; SE-W KO: 15.51 ± 0.63; EE-W WT: 15.23 ± 0.63; EE-W KO: 15.14 ± 0.63; Fig. 4*A*, middle) or adult (SE-A WT: 14.78 ± 0.54; SE-A KO: 15.2 ± 0.58; EE-A WT: 14.59 ± 0.54; EE-A KO: 15.03 ± 0.58; Fig. 4*A*, right) groups.

Omnibus tests showed a significant effect of housing (*F*_(1,3)_ = 16.971, *p* = 0.026) with a detectable interaction between housing and age (housing × age: *F*_(2,6)_ = 7.633, *p* = 0.022). Pairwise comparisons revealed that the decrease in the percentage of the dLGN occupied by ipsilateral terminals in EE-B KOs compared to SE-B KOs was significant (*p* < 0.001; Fig. 4*A*) as was the change in EE-B WTs compared to SE-B WTs (*p* < 0.001). SE-B WTs also had significantly more area of the dLGN occupied by ipsilateral terminals than SE-A WTs, suggesting a slight but significant age-dependent reduction in standard-housed mice (*p* = 0.003). A similar trend was seen when comparing SE-B KOs to SE-A KOs although this did not reach significance (*p* = 0.059). Importantly, the reduction in total ipsilateral terminal area seen in EE-B KOs was significant when compared to both EE-W KOs (*p* = 0.021) and EE-A KOs (*p* = 0.023) supporting a strong age-dependent effect of EE in KOs. EE-B WT values were no different from that of EE-W WTs (*p* = 0.901) or EE-A WTs (*p* = 1.000).

Thus, it appears that EE during the first six postnatal weeks may enhance (in KOs) or accelerate (in WTs) the pruning of ipsilateral projections. To determine whether this EE induced decrease in retinogeniculate terminal area was associated predominantly with a specific part of the dLGN, we divided the coronal cross-sections of the visual relay nucleus into thirds along its long (DM-VL) axis, and determined the relative distribution of ipsilateral terminals across these regions (Fig. 4*B–D*).

Quantitative analysis revealed a significant interaction between genotype, housing, age and region (*F*_(4,12)_ = 5.064, *p* = 0.013). No changes were seen in the relative distribution of ipsilateral terminals along the DM-VL axis of the dLGN as a result of age or housing for WTs (Fig. 4*B–D*). For KOs, a significant decrease was seen in the proportion of ipsilateral terminals which occupied the ventral-most third of the dLGN in EE-B KOs compared to SE-B KOs (*p* < 0.001; Fig. 4*B*). This was offset by a significant increase in the proportion of the ipsilateral terminals which populate the dorsal third of the dLGN in EE-B KOs compared to SE-B KOs (*p* < 0.001; Fig. 4*B*).

There was a generalized and consistent effect of KO mice having a significantly greater percentage of ipsilateral terminals present in the ventral-most region of the dLGN for weaning and adult age groups regardless of housing conditions (KO > WT: *p* ≤ 0.001; Fig. 4*C*,*D*), although this pattern did not hold for EE-B KOs. When considering specific comparisons, the proportion of terminals in the dorsal and ventral thirds of the dLGN for SE-B KOs was significantly different to SE-B WTs (*p* < 0.001; Fig. 4*B*,*D*). In contrast, for EE-B KOs versus EE-B WTs, there was no significant difference observed for dorsal dLGN (*p* = 0.158). An emergent difference (less in KOs) in the middle region, however, was detected (*p* = 0.003). Importantly, no difference between EE-B KOs and WTs was detected for ventral dLGN (*p* = 0.112) as well, suggesting an enrichment-induced reduction in ipsilateral terminals targeting this portion of the visual thalamus in EE-B KOs compared to SE-B KOs. Further, these EE-induced changes in the mapping of ipsilateral retinal axons were not seen in the weaning or adult KO groups for any area of the dLGN (*p* > 0.1; Fig. 4*B–D*).

**Figure 5.**
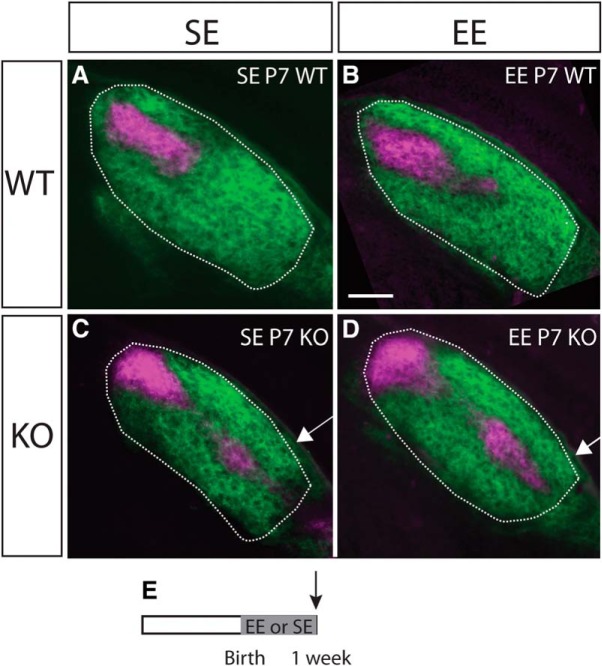
EE-B does not impact axonal targeting in Ten-m3 KOs at P7. Conventions are as for Figure 1, but showing examples from mice enriched from birth until P7, compared to standard-housed age-matched controls. Coronal sections through the rostral half of the dLGN (outlined) of mice following injections of anterograde fluorescent tracers into both eyes (dorsal is to the top and lateral to the right in all sections). No effect of EE on the targeting of ipsilateral retinal projections was apparent in WTs (***A***, ***B***) or Ten-m3 KOs (***C***, ***D***). Note ipsilateral label (magenta) extends into far ventrolateral dLGN in both EE KOs and SE KOs (arrows in ***C***, ***D***). ***E***, Schematic diagram indicating the time course of the experimental paradigm. The gray region indicates the period of exposure to EE versus SE. The arrow indicates the point of analysis. Scale bar in ***B*** = 100 μm, applies to all images.

The partial correction of mapping seen in the EE-B Ten-m3 KO mice following six weeks of EE could conceivably arise either due to an effect of enrichment on axonal targeting and ingrowth, a selective enhancement of synaptic pruning, or some combination of both. To gain more insight into the timing and potential processes activated by EE, we asked whether this manipulation impacted axon distribution at P7 in Ten-m3 KO mice. At this developmental stage, retinogeniculate axons of both standard-housed Ten-m3 KO and WTs have already invaded the dLGN, and are undergoing active synaptic refinement (Godement et al., 1984; Schafer et al., 2012; Hammer et al., 2014; Hong et al., 2014; Glendining et al., 2017; Monavarfeshani et al., 2018). Anterograde tracing revealed that ipsilateral axons extend into far ventrolateral dLGN in both SE and EE P7 KOs at this time point (Fig. 5). No difference was apparent between these groups though they both clearly differed from the terminal patterning observed in SE and EE WTs at this age. Quantification confirmed that there was no EE-induced change in the area of the dLGN occupied by ipsilateral terminals at this stage [SE P7 KOs (*n* = 5) vs EE P7 KOs (*n* = 5), housing as fixed and sections as random factors: *F*_(1,3)_ = 1.387; *p* = 0.324; housing × section interaction: *F*_(3,32)_ = 0.264, *p* = 0.780]. Similarly, there was no significant difference in the proportion of the ipsilateral terminals which targeted any region of the dLGN in EE versus SE P7 KOs at this stage of development (housing as fixed, and region as random factors: *F*_(1,2)_ = 0.000, *p* = 1.000; housing × region interaction: *F*_(2,114)_ = 0.475, *p* = 0.623).

Together, these results suggested that EE can induce a partial correction of axonal miswiring. Exposure to EE must commence in the first three postnatal weeks to have this effect. Although sensitivity to EE is high at this early stage, EE does not appear to impact axonal guidance or sprouting in the first postnatal week, but rather induces a selective pruning of mismapped ipsilateral retinal axons from the ventral region of the dLGN in Ten-m3 KOs. This enhanced pruning is not apparent by P7, but requires exposure to EE before P21 to induce the effect.

## Discussion

This study has revealed that EE-B, but not from weaning or during adulthood, is able to induce a partial correction of mismapped axonal projections. This appeared to be due to an enhanced pruning of ipsilateral retinal axons and predominantly affected the most aberrantly targeted projections. To our knowledge, this is the first study to demonstrate that EE can partially rescue genetically-determined axonal wiring deficits. These data suggest that EE has the capacity to exert a profound corrective influence on miswired subcortical circuits, but that there is an early critical period of sensitivity to this intervention.

### A critical period for the correction of miswired axonal projections

In contrast to EE commenced at birth which was able to induce a corrective influence on miswired axonal projections, EE commenced just three weeks later, at weaning, had no detectable impact. The dearth of an effect in the weaning group was particularly surprising, as the timing of EE exposure overlapped with the period when the retinogeniculate pathway is undergoing synaptic elimination and refinement. These findings suggest that the first three weeks of life may be a critical period in which the impact of EE on the retinogeniculate pathway is maximal. This contrasts with the impact of EE on the visual cortex where sensitivity to EE lasts throughout life (Sale et al., 2007; Baroncelli et al., 2010; Scali et al., 2012; Greifzu et al., 2016).

The first three postnatal weeks encompass the period during which retinal axons arrive in and form synaptic connections with their targets, ipsilateral and contralateral axons segregate from each other, the eyes open, and retinogeniculate synapses are pruned and mature (Godement et al., 1984; Hammer et al., 2014; Hong et al., 2014; Monavarfeshani et al., 2018). It is possible that EE is able to harness mechanisms associated with normal development to facilitate an enhanced elimination of mismapped projections.

The molecular mechanisms that underlie the impact of EE on the retinogeniculate pathway are not yet known. In the cortex, brain-derived neurotropic factor (BDNF) and IGF-1 have been shown to be upregulated by EE (Cancedda et al., 2004; Spires et al., 2004; Begenisic et al., 2015; Baroncelli et al., 2016). While BDNF is likely a key player, since it is also upregulated by EE in older mice (Sale et al., 2007; Baroncelli et al., 2010) it does not easily explain the results observed here, although it may be working in concert with other age-dependent factors. In line with this, while EE-induced cortical changes in both adults and juveniles involve upregulation of BDNF, downstream effects of the trophic factor appear to differ across the life span, and even have opposing effects on inhibitory circuits (Cancedda et al., 2004; Ciucci et al., 2007; Sale et al., 2007). Further work will be required to determine whether the temporally constrained influence of EE on the retinogeniculate pathway is due to the type of defect examined (i.e., impact of miswiring vs monocular deprivation) or if it reflects a genuine mechanistic difference in the response of cortical versus subcortical structures to EE.

EE for six weeks from birth resulted in an over 25% decrease in the size of the ipsilateral terminal zone in Ten-m3 KOs, predominantly from the most ventrolateral region of the dLGN. In contrast, exposure to EE-B to P7 had no apparent impact on retinal axon targeting. This suggests that EE acts not to correct initial axonal guidance or sprouting, but rather to enhance the subsequent pruning of ectopic projections. In standard-housed WTs, ipsilateral and contralateral retinal axons predominantly refine their territories via activity-dependent processes during the first and second postnatal weeks, before eye opening (Godement et al., 1984; Rossi et al., 2001; Jaubert-Miazza et al., 2005; Pfeiffenberger et al., 2005). The time course of ocular segregation in the dLGN of standard Ten-m3 KOs is identical to WTs despite their mismapped projections, suggesting that this refinement process is not disrupted (Glendining et al., 2017). An important role for microglia and the complement pathway in mediating ocular-refinement has been shown (Schafer et al., 2012; Bialas and Stevens, 2013). It is possible that mechanisms induced via EE-B may interact with this process, potentially by enhancing the activation of microglia in the ventral part of the dLGN in KO mice. Interestingly, the absence of a change in the area occupied by ipsilateral terminals in EE versus SE KOs at P7 suggests that ipsilateral-contralateral segregation was not accelerated by EE at this stage. It is possible that a longer exposure to EE may be required to induce an effect. Alternatively, a higher resolution analysis of overlap between ipsilaterally and contralaterally labeled pixels may be required to detect a change at this early stage, rather than percentage ipsilateral area as was used here.

### Subtle refinement of the retinogeniculate pathway continues into Adulthood in standard-housed mice, but is accelerated by EE

A slight but significant decline in the relative area occupied by ipsilateral terminals in the dLGN was found between the SE-B WT group which was analyzed at six weeks of age and the SE-A WT group (five to eight months old at analysis), with a similar but non-significant trend observed in standard-housed KOs. This change was surprising, as it is well-after the period during which this pathway is thought to mature (Jaubert-Miazza et al., 2005). This suggests that the period of pruning of retinogeniculate projections may continue beyond six weeks of age in standard-housed mice, albeit at fairly low levels. No differences were detected in EE-B WTs, compared to those enriched at later stages. Inspection of the values suggests that the ipsilateral refinement reaches adult levels earlier in EE versus SE WTs. Thus, it appears that EE accelerates this process such that age-related pruning is completed by six weeks of age, rather than extending beyond this time in SE WTs. No change was observed between the SE-A and EE-A groups, consistent with the suggestion that, in WTs, EE accelerates rather than enhances the degree of refinement per se.

### Comparison with other EE protocols

The critical period of exposure to EE for correction of mismapped inputs was found to include the pre-weaning period. Much of the impact of EE during these first postnatal weeks is thought to be derived from effects on maternal care (Cancedda et al., 2004; Begenisic et al., 2015; for review, see Sale et al., 2014), and this is likely to be important for the effects we observed here. Many EE protocols initiate enrichment postweaning and multiple beneficial effects have been reported (Rosenzweig et al., 1964; Bernstein, 1973; van Dellen et al., 2000; for review, see Sale et al., 2014). It is likely that EE commenced at later stages did affect Ten-m3 KO mice, although no impact on the parameters measured was observed.

Our EE protocol provided increased opportunities for visual, auditory and olfactory stimulation as well as enhanced motor and social experience. Other studies have indicated that the benefits of these components of EE may be partially separable (for review, see Sale et al., 2014; Rogers et al., 2019). It is unclear which of these factors are critical for the enhanced pruning of ectopic retinogeniculate projections observed in Ten-m3 KO mice. In particular, it would be of interest to determine whether visual experience is necessary, or whether the benefits of EE on the retinogeniculate pathway in Ten-m3 KOs can occur independently of vision (e.g., under dark rearing conditions) as revealed previously (Bartoletti et al., 2004; Landi et al., 2007).

### Behavioral significance

Standard-housed Ten-m3 KO mice have been reported to exhibit marked visual deficits associated with a suppression of binocularly-driven activity in V1. Since acute monocular inactivation lifts V1 suppression and rescues visual function, it has been suggested that the deficits arise due to the visuotopic mismatch of inputs from the two eyes (Leamey et al., 2007; Merlin et al., 2013). It would be of interest to determine whether the observed partial correction of the anatomical miswiring following EE-B is also able to drive the recovery of binocularly driven vision. Although only a partial correction was detected here, given the large size of visual receptive fields of RGCs in mice (Stone and Pinto, 1992), combined with thalamocortical and intracortical plasticity (Antonini et al., 1999; Sale et al., 2014), this may be sufficient to restore binocular visual drive to V1.

### Implications of findings

Our work has implications for the development of non-invasive therapies in humans. Enrichment paradigms that provide enhanced sensory experiences in autistic children have been shown to provide a significant decrease in the severity of symptoms (Woo and Leon, 2013; Woo et al., 2015; Aronoff et al., 2016), although no clear age-dependent effects were reported in these studies. Interestingly, other work has shown that behavioral interventions for autism have a much greater effect if they are initiated before the age of two years compared to slightly later ages (MacDonald et al., 2014). Our study provides clear, strong, anatomical evidence of distinct benefits for earlier versus later exposure to EE.

## Conclusion

This study has demonstrated that EE is able to drive the partial correction of aberrantly wired axonal projections. Importantly, this effect was only seen if EE was commenced in the perinatal period. This suggests that in addition to its previously-reported ability to modulate cortical plasticity across the life span, during an early critical period, EE can also drive anatomical repair of profoundly miswired subcortical circuits.

## Synthesis

Reviewing Editor: Tatiana Pasternak, University of Rochester Decisions are customarily a result of the Reviewing Editor and the peer reviewers coming together and discussing their recommendations until a consensus is reached. When revisions are invited, a fact-based synthesis statement explaining their decision and outlining what is needed to prepare a revision will be listed below. The following reviewer(s) agreed to reveal their identity: Ania Majewska.

The revised manuscript has adequately addressed the remaining reservations raised by the reviewers.
